# Supplementary material for: Dissecting Gap Junctional and Ephaptic Contributions to Electrical Conduction in a Novel Cardiomyocyte Pair Model
Source: bioRxiv. 2026 Mar 6:2026.03.05.709926. Preprint. [Version 1] doi: 10.64898/2026.03.05.709926 (PMC12970497; doi:10.64898/2026.03.05.709926)
Supplement: Supplementary file 1 [file media-1.pdf]

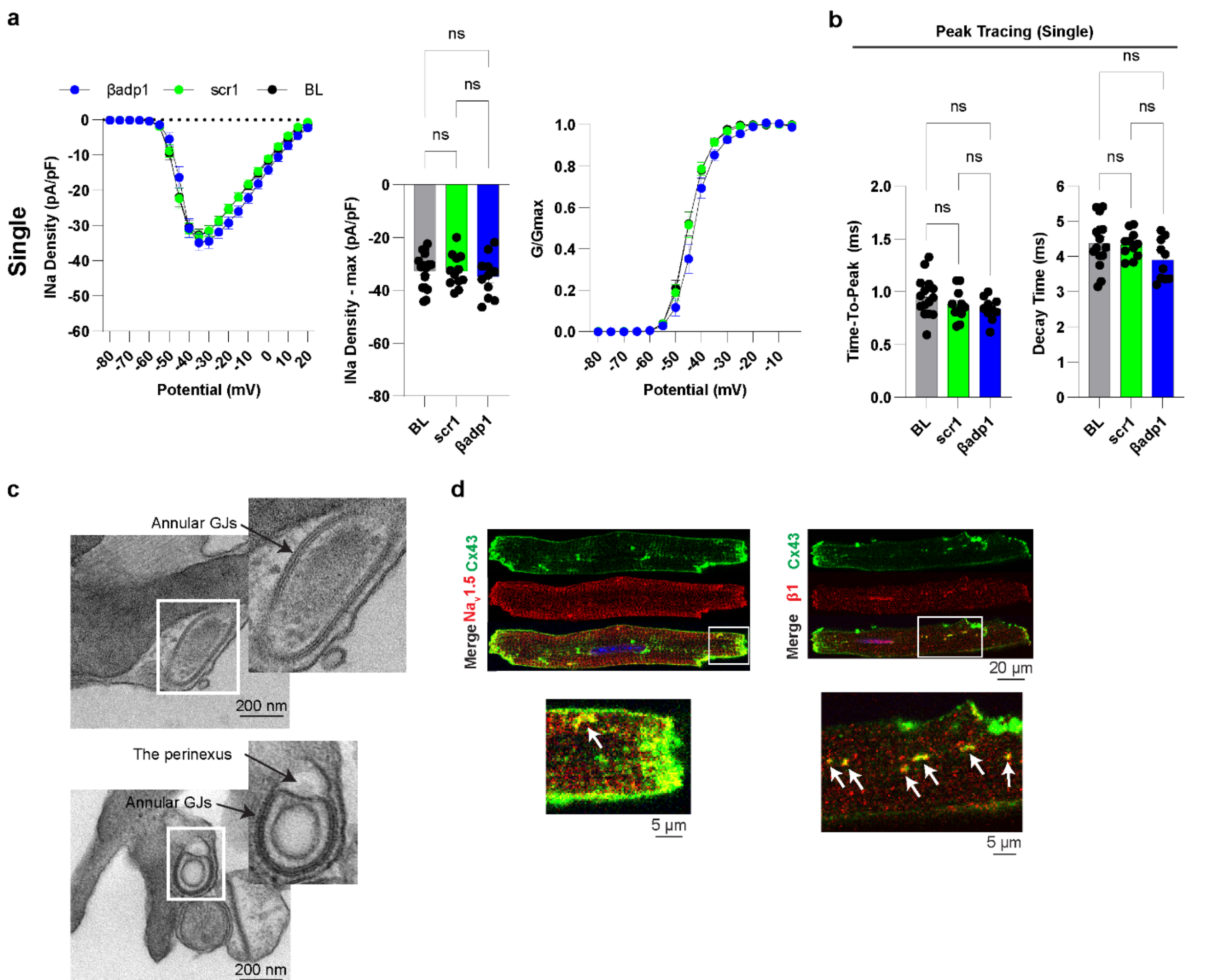

Figure S1. Perinexal remodeling increases sodium current in paired cardiomyocytes (continued). a. INa density I-V curves, max INa densities and steady-state activation curves in single cardiomyocytes at BL (n=17), scr1 (n=13) and βadp1 (n=11). **b**. Time-to-peak and decay time of the peak trace in single cardiomyocytes at BL, scr1 and βadp1. **c**. The internalization of gap junctions and perinexus near IDs in single cardiomyocytes. **d**. Immunofluorescent signals of Cx43&Nav1.5, Cx43&β1/β1b inside single cardiomyocytes. One-way ANOVA followed by Tukey correction for multiple comparison for **a** and **b**. *p<0.05; ns, not significant.

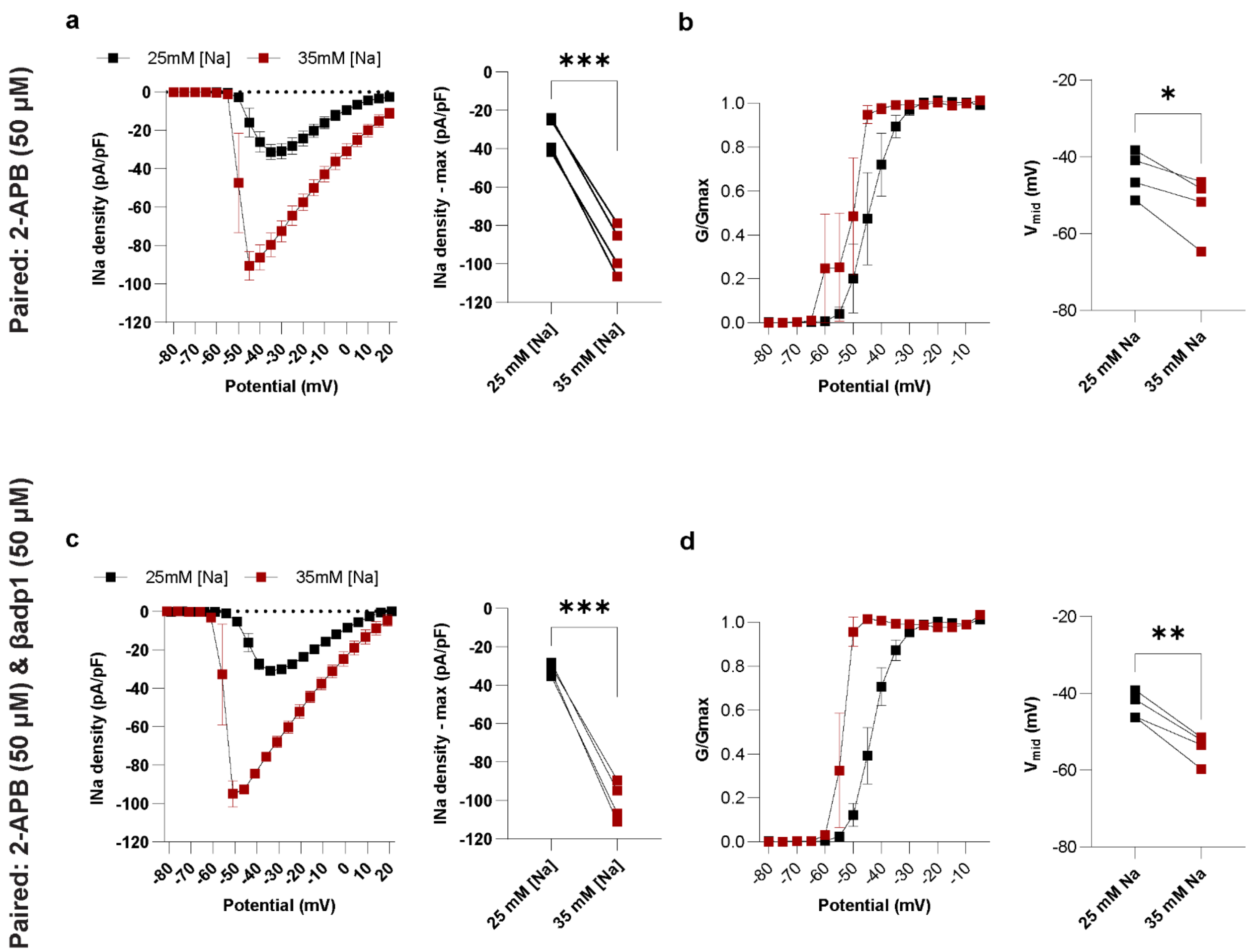
Paired: 2-APB (50 µM)
a
25mM [Na]
35mM [Na]
INa density (pA/pF)
Potential (mV)
INa density - max (pA/pF)
***
25 mM [Na]
35 mM [Na]
b
G/Gmax
Potential (mV)
V_mid (mV)
*
25 mM Na
35 mM Na
Paired: 2-APB (50 µM) & βadp1 (50 µM)
c
25mM [Na]
35mM [Na]
INa density (pA/pF)
Potential (mV)
INa density - max (pA/pF)
***
25 mM [Na]
35 mM [Na]
d
G/Gmax
Potential (mV)
V_mid (mV)
**
25 mM Na
35 mM Na

Figure S2. Increasing extracellular sodium restores the blocked electrical conduction between cardiomyocytes (*continued*). a. I_{Na} density I-V curves and max I_{Na} densities before and after acutely increasing extracellular sodium from 25 to 35 mM in same paired cardiomyocytes (n=4) which were treated with 50-μM 2-APB. **b**. Steady-state activation curves and V_{mid} before and after acutely increasing extracellular sodium from 25 to 35 mM in same paired cardiomyocytes (n=4) which were treated with 50-μM 2-APB. **c**. I_{Na} density I-V curves and max I_{Na} densities before and after acutely increasing extracellular sodium from 25 to 35 mM in same paired cardiomyocytes (n=4) which were treated with 50-μM 2-APB and 50-μM βadp1. **d**. Steady-state activation curves and V_{mid} before and after acutely increasing extracellular sodium from 25 to 35 mM in same paired cardiomyocytes (n=4) which were treated with 50-μM 2-APB and 50-μM βadp1.

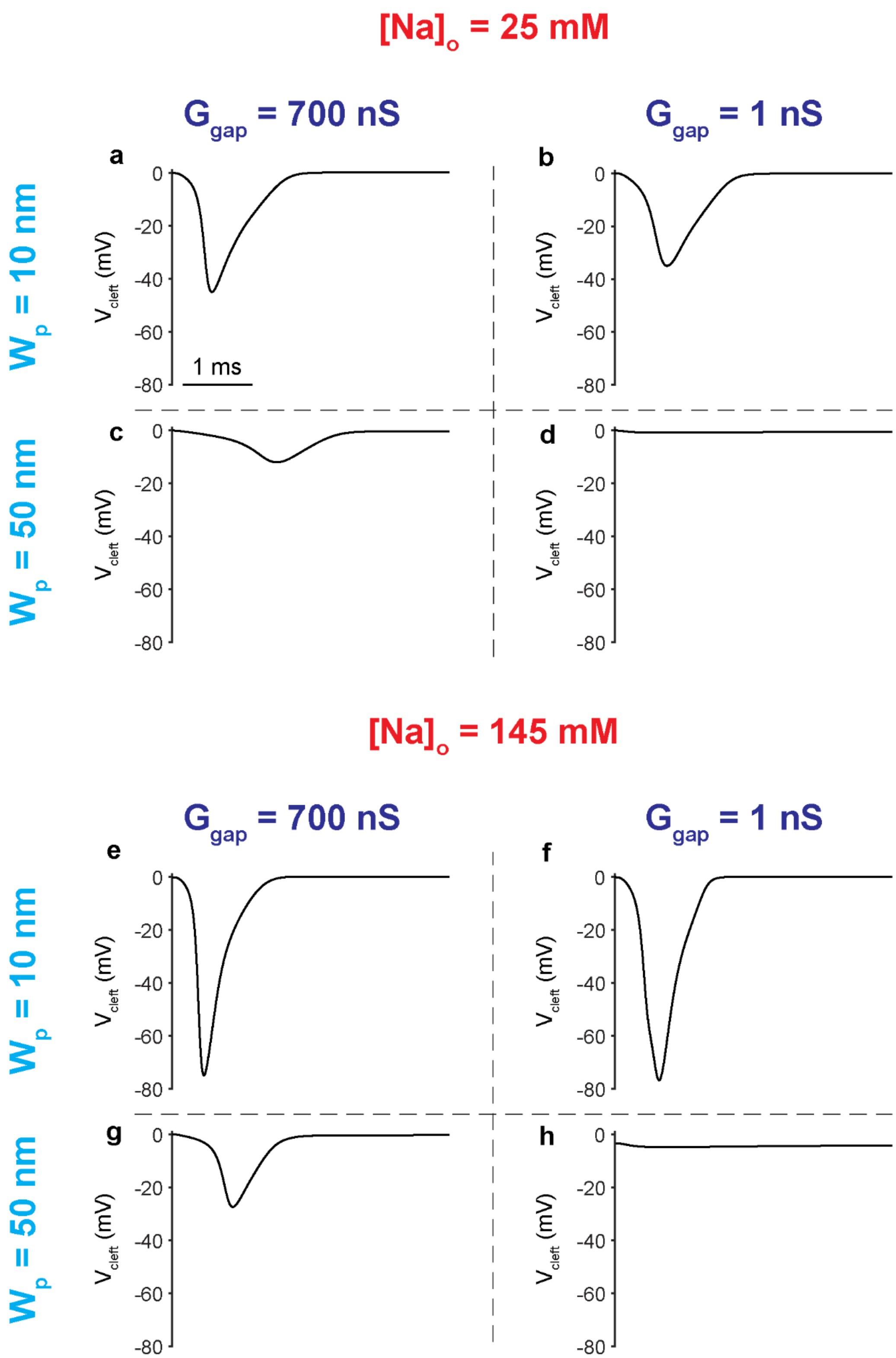

[Na]o = 25 mM
Ggap = 700 nS
Ggap = 1 nS
Wp = 10 nm
Wp = 50 nm
a
b
c
d
Vcleft (mV)
0
-20
-40
-60
-80
1 ms
[Na]o = 145 mM
Ggap = 700 nS
Ggap = 1 nS
Wp = 10 nm
Wp = 50 nm
e
f
g
h

Figure S3. Extracellular sodium concentrations determine the interplay between gap junctional and ephaptic coupling (*continued*). a, **b**, **c** and **d**: Cleft potentials under different strengths of GJC and EpC at a voltage step of -50 mV when the extracellular sodium is 25 mM. **e**, **f**, **g** and **h**: Cleft potentials under different strengths of GJC and EpC at a voltage step of -50 mV when the extracellular sodium is 145 mM.
